# Antibiotic Resistance Decreases the Efficacy of Endodontic Filling Pastes for Root Canal Treatment in Children′s Teeth

**DOI:** 10.3390/children8080692

**Published:** 2021-08-12

**Authors:** Claudia Adriana Rivera-Albarrán, Verónica Morales-Dorantes, José Luis Ayala-Herrera, Mariana Castillo-Aguillón, Uriel Soto-Barreras, Claudia Verónica Cabeza-Cabrera, Rubén Abraham Domínguez-Pérez

**Affiliations:** 1 Laboratory of Multidisciplinary Dentistry Research, Faculty of Medicine, Universidad Autónoma de Querétaro, Santiago de Querétaro 76176, Mexico; claudia.adriana.rivera@uaq.mx (C.A.R.-A.); veronicamoralesdorantes@gmail.com (V.M.-D.); 2 School of Odontology, Universidad De La Salle Bajío, León 37150, Mexico; jayala@delasalle.edu.mx; 3 Department of Pediatric Dentistry, Faculty of Medicine, Universidad Autónoma de Querétaro, Santiago de Querétaro 76176, Mexico; maryana_ca@hotmail.com (M.C.-A.); claudia.veronica.cabeza@uaq.mx (C.V.C.-C.); 4 Odontology Faculty, Universidad Autónoma de Chihuahua, Chihuahua 31125, Mexico; urielusb@gmail.com

**Keywords:** antibiotic resistance, deciduous teeth, root canal medicaments, *Streptococcus mutans*, *Enterococcus faecalis*

## Abstract

The antibacterial efficacy of antimicrobial filling pastes (AFP) used in the root canal treatment of primary teeth has been widely reported. However, antibiotic resistance as an emerging global problem could impact their current efficacy. This study aimed to evaluate the efficacy of two common AFP on susceptible or resistant bacteria isolated from primary necrotic molars. Microbiological samples were obtained and cultured from the root canals of 34 children. In total, 96 colony-forming units were obtained to determine their resistance to tetracycline, rifampicin, and chloramphenicol. They were identified as *S. mutans* or *E. faecalis* using polymerase chain reaction. The antimicrobial activity of CTZ paste (chloramphenicol, tetracycline, zinc oxide, and eugenol) and Guedes-Pinto modified (GPM) paste (rifampicin, prednisolone, iodoform, and camphorated paramonochlorophenol) were tested against the identified and selected microorganisms. Larger size inhibition zones were observed in both species when the tested strains were susceptible to the antibiotics in the AFP preparation. The efficacy of AFP containing antibiotics depends on the antibiotic resistance profile of the strain. Antibiotic resistance and its effect on the AFP were shown, which calls into question the use of simplified endodontic techniques that depend on antibiotics, since in these cases these techniques could not clinically eliminate resistant bacteria from the root canal.

## 1. Introduction

Endodontic treatment in primary teeth is indicated when the pulp has irreversible damage. This therapy is clinically challenging, and its success in pediatric dentistry is directly influenced by the elimination of the microorganisms in infected root canals. However, several complications could happen during the treatment, principally related to the complex morphology characterized by a marked root curvature, the interconnecting canals with side branches, junctions or apical ramifications, and physiological root resorption process, in addition to the lack of cooperation from children in most cases [1,2]. With these precedents, the use of antimicrobial materials has been proposed as one of the most important aspects of therapy in primary teeth [3].

Since the 1960s, the use of antimicrobial filling pastes (AFP) containing antibiotics such as chloramphenicol and tetracycline, in addition to zinc oxide and eugenol (CTZ paste), was proposed [4] for the treatment of primary teeth with necrotic pulp. In 1981, Guedes-Pinto formulated an AFP composed of iodoform, camphorated paramonoclorophenol, and Rifocort^®^ (an association of sodium rifamycin with corticosteroid) [5]. In the 1990s, the term “lesion sterilization and tissue repair” (LSTR) was proposed to refer to endodontic therapies using drug combinations to eliminate or minimize the number of microorganisms in the root canal system with necrotic pulps [6]. The objective of LSTR is to repair damaged tissues by disinfecting the affected pulp and periapical regions with a combination of antibiotics (generally the 3Mix paste, which is composed of metronidazole, ciprofloxacin, and minocycline) [7] and no mechanical instrumentation [8], regardless of pulp diagnosis [9]. This results in a less invasive treatment principally by avoiding the use of endodontic files, which decreases working time, finishing the procedure in a single appointment [10].

Some studies have reported high success with the LSTR technique for endodontic treatment of primary teeth [8,11], and clinical results have justified its use [12]. Nevertheless, some studies have reported low success rates in the radiographic assessment of the outcomes, confirming that unsuccessful treatments are commonly associated with symptom-free teeth [13,14].

The evaluation of antimicrobial effectiveness of a great diversity of AFP, using reference bacteria strains or bacteria obtained from clinical samples, has been performed for decades. As expected, when an AFP contains antibiotics, it has a greater effect. However, to date, it is considered subjective, since it is well known that the same bacterial genus or species could have different virulence and resistance factors. Furthermore, antibiotic resistance is much more prevalent today than it was just a few decades ago. Currently, treatments do not necessarily respond to the first-choice antibiotic, and theoretically, the same would be expected for AFP with antibiotics, meaning that these will no longer be effective in the short term.

The objective of this study was to evaluate the antimicrobial efficacy of two common antibiotic-based AFP on susceptible or resistant (to antibiotics used to prepare the tested AFP) strains isolated from root canals of primary necrotic molars. The hypothesis tested was that there is a difference between the antimicrobial efficacy of AFP in the same bacterial species depending on its antibiotic profile.

## 2. Materials and Methods

This mixed cross-sectional in vitro study included 34 children between 6 to 10 years old who came to request the attention of the pediatric dentistry department of the dental clinic at the faculty of medicine of the Autonomous University of Querétaro.

The study was approved by the ethical committee of the faculty. Informed written consent was obtained from either parents or guardians and the children assented to participate before the clinical examination. This consent embodied the ethical principles of the Declaration of Helsinki (Version 2008). A questionnaire about systemic and oral health status was completed by the children and their respective parent or guardian. None of the patients showed relevant information during the medical anamnesis.

The inclusion criteria comprised patients showing positive behavior with at least one primary molar with pulp necrosis diagnosis, a maximum of 1/3 of the root length, physiological resorption, the presence of fistulae or a radiolucent inter-radicular zone, and sufficient periodontal support which leads to low mobility and maintains the tooth as a functional one until the time of exfoliation. Exclusion criteria included antibiotic consumption during the last 6 months and difficulties in behavioral control during dental treatment.

### 2.1. Microbiological Sampling

After local anesthesia and rubber dam isolation, the operative field was cleaned with 3% hydrogen peroxide followed by 2.5% NaOCl solution. Dental caries removal and access preparation was performed using a sterile bur. Sterile saline solution was used for irrigation until the root canal entrances were visualized. A sterile paper cone (ISO 35) was inserted into the canals to a maximum of 1 mm from the apical limit, as measured from the initial radiograph. The paper cone was kept inside the canal for 1 min and was aseptically transferred to a microtube containing 1 mL of sterile phosphate-buffered saline (PBS). Only one molar was sampled from the child. After sampling, the teeth were instrumented and filled with calcium hydroxide paste, and a radiograph was taken. The provisional or definitive restoration were carried out depending on the clinical scenario.

### 2.2. Antibiotic Susceptibility Test

Each one of the 34 microtubes was vortexed and 50 µL was distributed in two agar plates, one containing brain–heart infusion (BHI) and the second, trypticase soy with sucrose and bacitracin (a selective medium for oral Streptococci). The agar plates were incubated at 37 °C for 24 to 48 h. In total, 96 colony-forming units (CFUs) were selected and inoculated in different culture tubes containing 5 mL of sterile BHI, and incubated for 24 h.

The Kirby–Bauer disc diffusion method determined three antibiotic resistance profiles. Tetracycline (30 µg), rifampicin (5 µg), and chloramphenicol (30 µg) discs (Oxoid, Basingstoke, UK) were applied onto the plates and incubated at 37 °C for 16 to 18 h. Resistance was considered when there was a zone of inhibition of ≤12 mm for chloramphenicol, ≤16 mm for rifampicin, and ≤14 mm for tetracycline based on the National Committee for Clinical Laboratory Standards (NCCLS) [15]. Out of the 96 CFUs, 38 (39%) were resistant to at least one of the three antibiotics (Table 1).

### 2.3. Microbial Identification and Selection of Strains of Interest

The 38 resistant strains plus 15 randomly chosen susceptible strains were submitted for molecular identification. Deoxyribonucleic acid (DNA) was extracted by phenol-chloroform purification and the isopropanol precipitation method. Polymerase chain reaction (PCR) assays were carried out in 25 µL of reaction using two different pairs of oligonucleotides: 5′-GGC ACC ACA TTG GGA AGC TCA GTT-3′ plus 5′-GGA ATG GCC GCT AAG TCA ACA GGA T-3′ for *S. mutans* [16] and 5′-TAC TGA CAA ACC ATT CAT GAT G-3′ plus 5′-AAC TTC GTC ACC AAC GCG AAC-3′ for *E. faecalis* [17]. PCR products were analyzed by electrophoresis in a 2% agarose gel and a 100-bp DNA ladder marker. Each gel was stained with ethidium bromide and observed under ultraviolet light. Positive reactions were determined by bands of the expected sizes. Six resistant strains were identified, three as *S. mutans* and three as *E. faecalis*, as well as three susceptible strains, one as *S. mutans* and two as *E. faecalis.* The nine strains were included in the AFP susceptibility test.

### 2.4. Antimicrobial Filling Paste Susceptibility Test

The antimicrobial activity of the two common AFP was tested against previously selected strains. Since Ricofort^®^ is no longer available, a modification of the Guedes-Pinto (GPM) paste was used. It was prepared with 0.25 mL of rifampicin, 5 mg of prednisolone, 300 mg of iodoform, and 0.1 mL of camphorated paramonochlorophenol. The CTZ paste was prepared with 500 mg of chloramphenicol, 500 mg of tetracycline chloride, 1000 mg of zinc oxide, and 150 µL of eugenol. Both pastes were prepared with a toothpaste consistency. Ninety plates with 20 mL of BHI agar were inoculated with 0.1 mL of each microbial suspension (10 plates for each one). Monolayer growth was obtained by spreading the culture with sterile swabs on the agar. Four paper discs of 6 mm in diameter were charged in each plate, one for the GPM paste and the other for the CTZ paste. Additionally, a positive control with chlorhexidine and a negative control with distilled water were included. The plates were pre-incubated for one hour at room temperature and then incubated at 37 °C for 24 to 48 h. Additionally, positive and negative controls were carried out, keeping the plates inoculated and without inoculum for the same period. All assays were carried out under aseptic conditions. Zones of bacterial inhibition and material diffusion were measured in millimeters using a digital caliper (Mitutoyo, São Paulo, SP, Brazil). Measurements were taken at the largest distance between two points at the outer limit of the inhibition zone around the paper disc.

### 2.5. Statistical Analysis

Antibiotic susceptibility tests were repeated three times to confirm the homogeneity of the results. The AFP test results are expressed as means and standard deviation, and were subjected to statistical parametric tests of two-way ANOVA and post hoc Tukey–Kramer multiple comparisons test using Graph-Pad Instat, version 3.0 (Graphpad Software, San Diego, CA, USA). Statistical significance was set at *p* < 0.05.

## 3. Results

**Figure 1:**
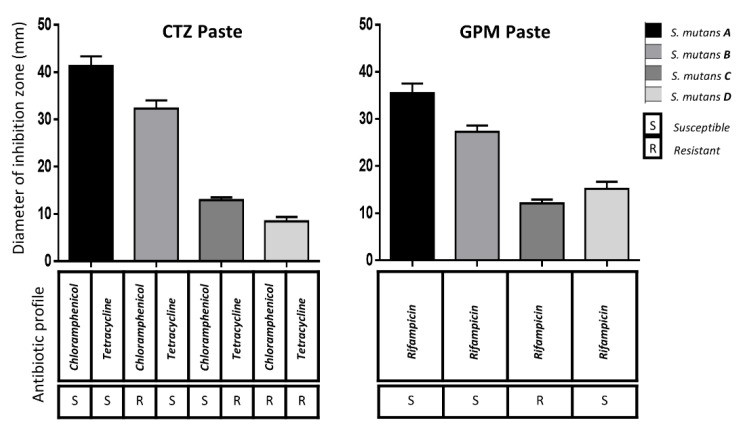
Average and standard deviation of the inhibition zones on the four different *S. mutans* strains with the use of CTZ and GPM pastes. Under the bars, the specific antibiotic profile of each strain is shown. All the comparisons showed differences (*p* < 0.05). Two-way ANOVA and post hoc Tukey–Kramer multiple comparisons test were applied.

**Figure 2:**
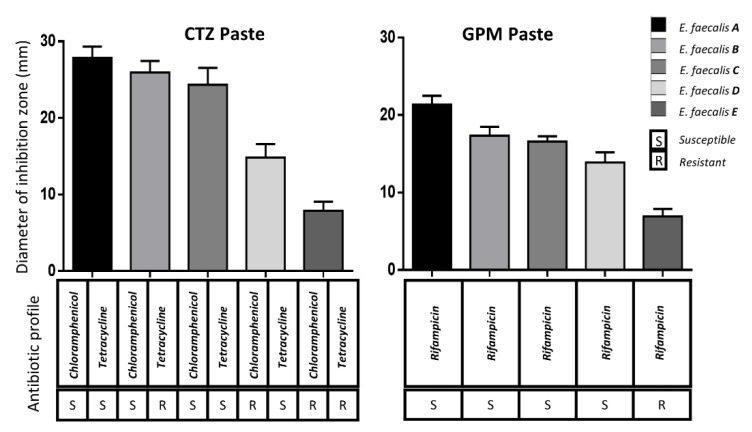
Average and standard deviation of the inhibition zones on the five different *E. faecalis* strains with the use of CTZ and GPM pastes. Under the bars, the specific antibiotic profile of each strain is shown. The comparisons with no differences (*p* > 0.05) were the strains A vs. B and B vs. C when CTZ paste was used, and the strains B vs. C when GPM paste was used. Two-way ANOVA and post hoc Tukey–Kramer multiple comparisons test were applied.

The results of the AFP susceptibility test are shown in Figure 1 for *S. mutans* and Figure 2 for *E. faecalis*. The size difference of the inhibition zones was evident (*p* ≤ 0.05) when the antibiotic profile of the tested strain was susceptible or resistant to the specific antibiotic contained in the tested AFP.

There were differences (*p* ≤ 0.05) when the results of the *S. mutans* strains or *E. faecalis* were compared. The only comparison with no difference (*p* > 0.05) using CTZ paste was when comparing *E. faecalis* A versus *E. faecalis* B. The comparison of the *E. faecalis* B versus C strains using GPM or CTZ pastes did not show differences (*p* > 0.05), as both cases had a very similar antibiotic profile.

The CTZ paste was superior to the GPM paste in most cases. However, there were three cases with no significant differences (*S. mutans C*, *E. faecalis D,* and *E. faecalis E*) and one case (*S. mutans D*) in which the GPM paste was superior to CTZ. In each case, the chlorhexidine zone was ≥30 mm, while the distilled water control was ≤8 mm.

## 4. Discussion

There are different techniques and protocols for pulp treatment in primary teeth, which depend on the extent of the damage and the pathological compromise [18,19]. Endodontic principles recommend absolute isolation, odontometry, chemical–mechanical cleaning, and filling to eradicate bacteria when a pulpectomy is performed. However, several complications could occur during the treatment. These complications have led clinicians to propose simplified techniques [20] based on AFP use, including antibiotic preparations. Antibiotic resistance has become a widespread menace that requires attention and intervention. It is of the utmost importance, because it limits the therapeutic options for effective treatment against infections. The most severe consequence is an antibiotic failure. It complicates the treatment, risks the patient’s life, and increases the economic investment of global medical care. The intensive use of antibiotics in medicine, dentistry and non-medical settings, such as animal farming and agriculture, are the main reasons for the rapid increase in antibiotic resistance [21]. Diverse bacterial resistance mechanisms to antibiotics are transferred by genetic material, known as resistance genes, which have been fully described and identified for many antimicrobial agents [22]. The oral microbiota has been described as a reservoir for several antibiotic resistance genes [23]. Like other multi-species biofilms, oral bacterial species are arranged in proximity, which frequently leads to interactions such as quorum-sensing systems, food chains, and exchange of virulence and resistance genes [24], including endodontic biofilms involved in pulp and periapical pathologies [25].

*S. mutans* and *E. faecalis* were selected as representative bacteria from the endodontic biofilm in this study. However, other species were collected, cultured, isolated, and tested to establish their antibiotic profile. A high prevalence (39%) of bacteria with resistance to the tested antibiotics was found. Even though parents confirmed that they had never administered chloramphenicol or rifampicin to their children, it was alarming to find three resistant strains to these antibiotics. The presence of resistant strains in the children could be because of the transfer of resistant bacteria by their mothers or their pets [26]. Although this study only included a number of limited species, it did include multiple strains for each species with different antibiotic profiles, allowing the differentiation between the antimicrobial efficacies of the AFP. The susceptible strains showed greater inhibition zones compared with the low or null inhibition zones of the resistant strains. Thus, the hypothesis had to be accepted.

The CTZ paste was predominantly superior to the GPM paste. This could be due to the enhanced antimicrobial effect in CTZ paste of its two broad-spectrum antibiotics (tetracycline and chloramphenicol), compared to GPM paste, which only contains one antibiotic (rifampicin). However, in some cases, there were no significant differences between both pastes. Even when *S. mutans* was resistant to tetracycline and chloramphenicol, the GPM paste showed a major effect, which could be explained by its camphorated paramonochlorophenol as an antimicrobial compound.

Regarding the CTZ paste, differences in the inhibition zones for strains with a mixed antibiotic profile (*S. mutans* B and C, and *E. faecalis* B and D) were observed. Tetracycline was more effective than chloramphenicol in *S. mutans* strains, since the inhibition zone was greater in strain B compared with strain C. On the other hand, chloramphenicol was more effective than tetracycline in *E. faecalis*, since the inhibition zone was greater in B compared with D strains.

The attempt to evaluate the antimicrobial effectiveness of AFP with antibiotics has become unavailing. The results over reference strains or clinical isolates cannot be clinically used. The effect of an antibiotic on a bacterial genus or species is becoming more and more unpredictable. It must be kept in mind that even though a strain belongs to the same species, it could have different virulence factors and antibiotic resistance.

Antibiotic resistance and its effect on the AFP were shown in this study, which calls into question the effectiveness of endodontic techniques that rely on the antibiotic effect to avoid chemical–mechanical cleaning and disinfection of the pulp canal, since they could not clinically eliminate resistant bacteria. On the other hand, the use of AFP with antibiotics also promotes antibiotic resistance and the beginning of a vicious circle. Although there are no studies that prove that AFP cause resistance, there is evidence that the use of topical antibiotics does cause it. Studies in dermatology [27] or ophthalmology have described that repeated exposure to ocular antibiotics increases multidrug resistance [28].

The responsible use of antibiotics is necessary. As clinicians, it is necessary to decide if we want simple, fast, non-invasive procedures that save resources and time, even if these procedures will gradually become outdated and contribute to greater antibiotic resistance as a global problem. It is time to return to instrumented endodontic techniques, even though this implies more economical cost, working time, and clinical training. Additionally, it is important to develop and evaluate new materials that eliminate bacteria through different antimicrobial mechanisms that do not trigger resistance, or increase the use of calcium hydroxide or zinc oxide eugenol fillings, which have been shown to reduce bacteria in primary root canals and provide long-term radiographic success [14,29].

## 5. Conclusions

Antibiotic resistance has become a widespread problem. The efficacy of AFP containing antibiotics has decreased because of the high prevalence of resistant bacteria in the root canals. It was shown that the AFP efficacy depends on the antibiotic resistance profile of the present bacteria.

## Figures and Tables

**Table 1:** Frequency and percentage of resistance to the accessed antibiotics in the 96 cultures.

	Cultures (*n* = 96)
Chloramphenicol	7 (7.2)
Tetracycline	9 (9.3)
Rifampicin	5 (5.2)
Chloramphenicol + Tetracycline	6 (6.2)
Chloramphenicol + Rifampicin	2 (2.0)
Tetracicline + Rifampicin	6 (6.2)
Chloramphenicol + Tetracycline + Rifampicin	3 (3.1)
Without resistance (for these antibiotics)	58 (60.4)

## Data Availability

The dataset used and analyzed during this study is available from the corresponding author on reasonable request.
